# Phylogenomic analysis reveals splicing as a mechanism of parallel evolution of non-canonical SVAs in hominine primates

**DOI:** 10.1186/s13100-018-0135-2

**Published:** 2018-09-17

**Authors:** Annette Damert

**Affiliations:** Primate Genetics Laboratory, German Primate Center, Leibniz Institute for Primate Research, Göttingen, Germany

## Abstract

SVA (SINE-R-VNTR-*Alu*) elements are non-autonomous non-LTR (Long Terminal Repeat) retrotransposons. They are found in all hominoid primates but did not amplify to appreciable numbers in gibbons. Recently, phylogenetic networks of hominid (orangutan, gorilla, chimpanzee, human) SVA elements based on comparison of overall sequence identity have been reported. Here I present a detailed phylogeny of SVA_D elements in gorilla, chimpanzee and humans based on sorting of co-segregating substitutions. Complementary comparative genomics analysis revealed that the majority (1763 out of 1826–97%) of SVA_D elements in gorilla represent species-specific insertions – indicating very low activity of the subfamily before the gorilla/chimpanzee-human split. The origin of the human-specific subfamily SVA_F could be traced back to a source element in the hominine common ancestor. The major expanding lineage-specific subfamilies were found to differ between chimpanzee and humans. Precursors of the dominant chimpanzee SVA_D subfamily are present in humans; however, they did not expand to appreciable levels.

The analysis also uncovered that one of the chimpanzee-specific subfamilies was formed by splicing of the *STK40* first exon to the SVA *Alu*-like region. Many of the 94 subfamily members contain additional 5′ transductions – among them exons of 8 different other genes. Striking similarities to the *MAST2*-containing human SVA_F1 suggest parallel evolution of non-canonical SVAs in chimpanzees and humans.

**Electronic supplementary material:**

The online version of this article (10.1186/s13100-018-0135-2) contains supplementary material, which is available to authorized users.

## Background

**Fig. 1:**
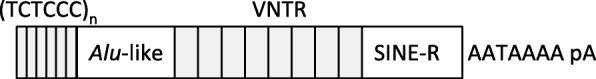
Schematic representation of an SVA element. The element consists of (from 5′) TCTCCC hexameric repeats, an *Alu*-like domain, a VNTR (Variable Number of Tandem Repeats) region and an endogenous retrovirus-derived SINE-R at the 3′. The polyadenylation signal is directly followed by the polyA tail (pA)

SVAs (SINE-R-VNTR-*Alu*, Fig. 1) are composite non-autonomous non-LTR (Long Terminal Repeat) retrotransposons consisting of a 5′ hexameric repeat region followed by antisense *Alu* copies (*Alu*-like), a VNTR (Variable Number of Tandem Repeats) domain and a 3′ region derived from an endogenous retrovirus (SINE-R). SVA elements are present in all hominoid primates (that is gibbons and great apes [1]). However, they did not amplify to appreciable numbers in gibbons. SVAs are mobilized by the proteins encoded by their autonomous partner element LINE-1 (Long Interspersed Element; L1) in *trans* [2, 3].

Retrotransposons evolve as hierarchical subfamilies. Mutations accumulated in a source element are passed on to its offspring during retrotransposition. They are shared by all members of the subfamily. The number of subfamily-specific shared mutations increases from evolutionary older towards younger subfamilies. Conversely, random mutations acquired after insertion are more frequent in older subfamilies and less abundant in the younger ones (regarding *Alu* subfamilies see [4] and references therein). Shared subfamily-specific mutations are used as diagnostic substitutions in the identification of retrotransposon subfamilies (see e.g. [5]).

SVA subfamilies were first characterized by Wang and colleagues in 2005. Age estimates indicated that SVA_A (the evolutionary oldest subfamily) “may have expanded contemporary to the divergence of the orangutan and the great apes”. For SVA_B, SVA_C and SVA_D they suggested expansion predating the gorilla/chimpanzee/human split. SVA_E and SVA_F were found to be human-specific. Wang et al. also identified a chimpanzee-specific subfamily, which they named SVA_PtA [1]. In 2009 three groups independently characterized a non-canonical SVA_F subgroup in which the larger part of the 5’ *Alu*-like domain has been replaced by the first exon of the *MAST2* gene [6–8]. In the context of an analysis of the SVA VNTR domain 12 SVA subfamilies have been identified in the orangutan genome [9]. A detailed analysis of SVA elements in gorilla and chimpanzee had been missing until recently when Levy and colleagues published phylogenetic networks for SVA elements in all four hominids [10]. Their results are based on pairwise comparison of overall sequence similarity between elements. Subfamilies are identified using a network-based approach.

Two of their findings and resulting claims attracted my special interest: first, in their “all-hominid network” they found that “human SVA_E and SVA_F, which were thought to be human-specific, contained elements from the chimp and gorilla genomes, too” [10]. Second, based on their discovery of SVAs belonging to different subfamilies “in the exact same location in the human and orangutan genomes” in the *CABIN1* and *NPLOC4* genes they postulate the existence of an SVA “master element” active over a long period in evolution that consecutively gave rise to SVA subfamilies SVA_B to SVA_D [10].

Drawing on preliminary data for chimpanzee and gorilla available from an earlier study [9] and taking advantage of a substantially improved gorilla genome build that had become available [11] I decided to conduct a detailed phylogenetic analysis of SVA_D – the subfamily currently active in hominines and source of the human-specific subfamilies SVA_E and SVA_F – in gorilla, chimpanzee and humans.

The analysis presented here uses manual sorting of elements into subfamilies based on shared diagnostic substitutions inherited from a common ancestor. Combination of the subfamily analysis with comparative genomics confirmed the existence of subfamilies with shared source elements and independent expansion in the different species as suggested by Levy et al. [10]. In-depth analysis of the origins of the human SVA subfamily SVA_F found it dating back to a source element present in the hominine common ancestor.

Most surprisingly, the analysis uncovered a striking case of parallel evolution of non-canonical SVA elements in humans and chimpanzees: similar to the emergence of SVA_F1 in humans due to splicing of the *MAST2* first exon to the 5’ SVA *Alu*-like domain, chimpanzee subfamily pt_SVA_D6 has acquired the first exon of *STK40* and expanded to comparable copy numbers as SVA_F1 in humans.

## Results and discussion

### A detailed map of SVA_D expansion in hominines – Species-specific networks

**Fig. 2:**
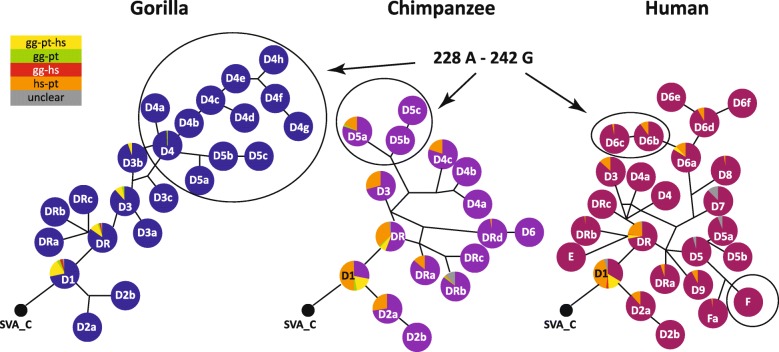
Phylogeny of SVA_D elements in hominines. Median-joining networks of the SVA_D SINE-R domains. Networks were rooted on SVA_C and are not drawn to scale. Elements shared between the lineages are shown as fraction of the total number of members of a subfamily. A color code for shared elements is provided at the left. Ovals indicate the major expanding subfamilies in the different lineages: SVA_D elements with characteristic 228 A – 242 G substitutions in gorilla and chimpanzee and SVA_F in humans. The part of the human network displaying the 228 A – 242 G substitutions is marked as well. SVA_E is included in the human network to comprehensively reflect the spectrum of currently active SVA elements. gg – gorilla; pt – chimpanzee; hs – human

In the first analysis of SVA subfamilies in the human genome Wang et al. [1] provide an age estimate of 9.55 myrs (million years) for subfamily SVA_D. In the context of the currently accepted split times for hominines [12] expansion of the subfamily would, thus, coincide with the gorilla/chimpanzee-human split. Detailed analysis of the subfamily suggests that there has been only limited amplification of SVA_D elements before the split: in gorilla there are only 47 SVA_D elements shared with humans, the majority of them are also present in chimpanzees. SVAs present in human and gorilla and absent in chimpanzees must have been lost in this lineage, either due to incomplete lineage sorting or, less likely, through precise deletion. Ten elements were found to be shared between gorilla and chimpanzees, being absent from the human genome. In total 1764 SVA_D elements are gorilla-specific, 1239 SVA_D elements are found specifically in the chimpanzee genome and 916 human-specific SVA_Ds bring the total human-specific population of SVAs (including subfamilies SVA_E and SVA_F) up to 1395 (Additional files 1 and 2). The numbers of lineage-specific insertions found for humans and chimpanzees are in accordance with those reported previously [13]. For gorilla Gordon et al. reported 1498 species-specific SVA elements based on mapping of contigs greater than 200 kbp to hg38 [11]. In median-joining networks constructed based on the SINE-R domains of the SVA_D elements (Fig. 2, positions and subfamily affiliations for all elements are provided in Additional file 2, consensus sequences for all subfamilies as Additional file 3), most of the elements shared across all three lineages are found in the most basal SVA_D subfamily, D1. Interestingly, D1 gave rise to subfamilies with a “trademark” 20 bp deletion in the SINE-R (D2) in all three lineages. The D2 source element that must have been present in the hominine common ancestor is no longer identifiable. Shared elements are still to be found in the homologous chimp and human D2a subfamilies. Sequence comparison of the consensus sequences (Additional file 3) suggests that the derived D2b families in these lineages also share a common ancestor. However, it can no longer be found. Assessment of presence/absence polymorphisms suggests that SVA_D2b elements are fixed in humans and chimpanzees (not shown), i.e. they have ceased to be active.

In addition to the closely related D2 subfamilies in chimpanzees and humans there are a number of other groups that comprise shared as well as lineage specific elements (Fig. 2) – indicating that expansion started before the split and independently continued in each of the species thereafter. Such a scenario is also suggested by the all-hominid network presented by Levy et al. [10]. The pairs of subfamilies identified here are: pt_D5a and hs_D6b, pt_D4c and hs_D6f, pt_D3/hs_D3 (D3 in [10]) and pt_DRa/hs_DRa. In another subfamily (hs_D6a) there are shared elements, however, in chimpanzees they do not appear to have formed a recognizable subfamily. Table 1 summarizes corresponding subfamilies in gorilla, chimpanzee and humans.

**Table 1:** Corresponding SVA_D subfamilies in gorilla, chimpanzee and human

Gorilla	Chimpanzee	Human	Observations
gg_SVA_D1	pt_SVA_D1	hs_SVA_D1	
gg_SVA_D2a			
gg_SVA_D2b			
	pt_SVA_D2a	hs_SVA_D2a	
	pt_SVA_D2b	hs_SVA_D2b	1 species-specific substitution each
gg_SVA_DR	pt_SVA_DR	hs_SVA_DR	
gg_SVA_DRa		hs_SVA_D9	
gg_SVA_DRb			shared substitutions with SVA_E
gg_SVA_DRc		hs_SVA_DRb	2 species-specific substitutions in hs
gg_SVA_D3			
gg_SVA_D3a		hs_SVA_D7	3 species-specific substitutions in hs
gg_SVA_D3b			
gg_SVA_D3c			
gg_SVA_D4	pt_SVA_D5a	hs_SVA_D6b	1 species-specific substitution each; identical in hs&pt
gg_SVA_D4a-h			
gg_SVA_D5a-c			
	pt_SVA_DRa	hs_SVA_DRa	
	pt_SVA_DRb	hs_SVA_Fa	2 species-specific substitution in pt; 1 in hs
	pt_SVA_DRc	hs_SVA_D5a	2 species-specific substitution in pt; 1 in hs
	pt_SVA_DRd	hs_SVA_D4	1 species-specific substitution in hs
	pt_SVA_D3	hs_SVA_D3	
	pt_SVA_D4a/b		see Fig. 3c
	pt_SVA_D4c	hs_SVA_D6f	
	pt_SVA_D5b/c		see Fig. 3c
	pt_SVA_D6		
		hs_SVA_DRc	
		hs_SVA_D4a	
		hs_SVA_D5	small number of elements in pt
		hs_SVA_D5b	
		hs_SVA_D6a, c, d, e	see Fig. 3c
		hs_SVA_D8	

Subfamilies listed in a single column are species-specific. Species-specific substitutions in the last column refer to substitutions present in addition to those shared across species. *gg* gorilla, *pt* chimpanzee, *hs *human

Comparison to the networks published and supplementary information provided by Levy et al. [10] reveals that the majority of the subfamilies identified here by the diagnostic residue-based sorting approach are recovered there as well – some of them at the initial resolution used for the comparative hominine networks, some as parts of communities in the all-hominid network (see [10] figures 2 and 4, respectively). A summary of the corresponding subfamilies is provided in Additional file 4: Table S1.

In two cases the diagnostic substitution-based sorting used here provides higher resolution than the network-based approach as presented in Levy’s all-hominid network (the network with the highest resolution provided in [10], their figure 4): the terminal part of gg_SVA_D4 (starting from gg_SVA_D4b in Fig. 2 and corresponding to SVA_D4_Gg) and the human hs_SVA_D6 complex (Fig. 2; found in the D1d/D5_Pt community in [10], for details see below).

A puzzling finding in Levy et al.’s all-hominid network is the placement of SVA_E. In their analysis ([10], their figure 4) a node that links D3 and E branches off from D1a (corresponding to gg/pt/hs_SVA_D1), the most basal SVA_D subfamily. D1a is separated from SVA_C by 12 substitutions; three additional substitutions were acquired on the path to subfamily DR. However, both D3 and E contain all 15 diagnostic substitutions characteristic for DR. Consequently, they should branch off from DR (D1b, c in [10] respectively). Closer inspection of the node community connecting D1a and E revealed that it comprises solely 5′ truncated elements of all three hominines. 11 out of 12 diagnostic residues distinguishing E from SVA_D are not covered by the majority of sequences; in the very few cases where the last two of them are covered the sequences do not correspond to the SVA_E consensus. This community should, therefore, not be considered as a node supporting the origin of SVA_E from D1a (gg/pt/hs_SVA_D1). As far as elements suggested to be shared between gorilla (gg_SVA_DRb) and human SVA_E are concerned, re-analysis by LiftOver and manual curation could not confirm that any of the elements present in the E community is shared between species. The most likely scenario for the emergence of gg_SVA_DRb and human SVA_E is therefore a source element in the hominine common ancestor corresponding in sequence to gg_SVA_DRb. The element itself was lost in all three hominines, however, in the human lineage only after giving rise to the founder element of SVA_E. Considering that gg_SVA_DRb and SVA_E share eight diagnostic substitutions, their independent evolution in gorilla and humans, respectively, is rather unlikely.

### Cross-species networks

**Fig. 3:**
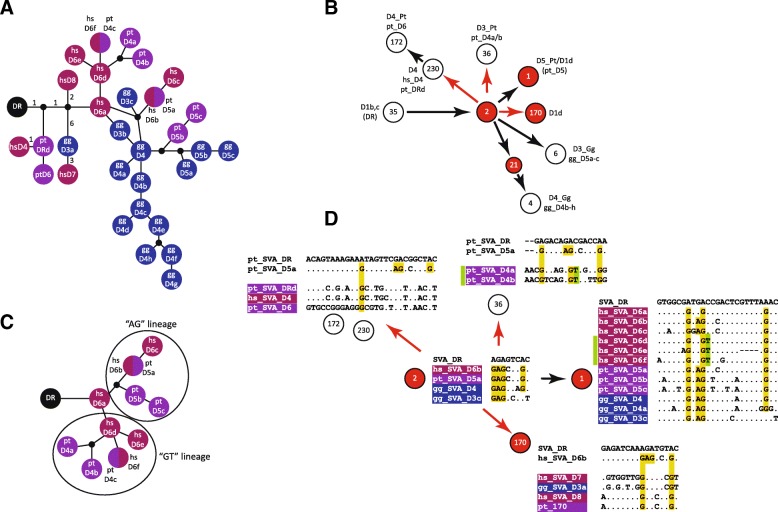
Comparison of all-hominine networks. **a** All-hominine network based on SVA_D subfamilies identified by diagnostic substitution-based sorting. Split circles represent subfamilies with identical consensus sequences. Numbers in the proximal part of the network indicate the number of substitutions between nodes. The network is not drawn to scale. **b** Corresponding part of the all-hominid network as presented by Levy et al. [10]. Communities are represented by circles and their reference number as determined from the data provided in the supplement. Subfamily names are those assigned by Levy et al. (on top) and in this study (below). **c** Human-chimpanzee cross-species network detailing the hs_D6/pt_D4 and pt_D5 part of the network shown in (**a**). The different lineages are marked by ovals. The network is not drawn to scale. **d** Alignments of consensus sequences of the constituent subfamilies of the communities shown in (**b**). SVA_DR is given as reference for all alignments. Only variant positions are shown. The alignments for communities 230/172, 36 and 170 in addition contain the consensus sequence of the subfamily present in the alleged precursor community 2 (pt_SVA_D5a or hs_SVA_D6b) for comparison. Shared diagnostic substitutions present in community 2 are highlighted in yellow; hs_SVA_D6d-f and pt_SVA_D4a-b specific shared substitutions are highlighted in green. Communities whose constituent subfamilies are discussed in the main text are highlighted in red in (**b**) and (**d**). Red arrows indicate phylogenetic relationships not supported by the network shown in (**a**)

In their recent publication Levy et al. (figure 4 in [10]) present an SVA all-hominid network based on which they draw conclusions about common origins and species-specific amplifications of SVA subfamilies across orangutan, gorilla, chimpanzee and humans. Having noticed inconsistencies between their network and my own analysis with regard to the placement of SVA_E, I generated an all-hominine network based on the subfamily consensus sequences recovered in my analysis and compared it to the one presented by Levy et al. [10]. As I had found the phylogenetic relationships in the proximal part of the network consistent with my own analysis, I focused on the distal part of the SVA_D network from D1b,c to the 96-member node and derived communities. This part of the network covers the major amplifying subfamilies in gorilla and chimpanzee as well as related human elements. The all-hominine network generated using the subfamily consensus sequences obtained by sorting based on diagnostic substitutions is shown in Fig. 3a, the corresponding part of Levy et al.’s network is provided in Fig. 3b. The roots of both networks (DR and D1b, c) are roughly comparable with regard to the elements they contain.

Sorting of the constituent elements of the communities defined by Levy et al. (Fig. 3d) revealed (a) incorrectly assigned phylogenetic relationships (red arrows), (b) split of subfamilies between communities 1 and 2 and (c) communities containing subfamilies expected to be separated based on the pattern of diagnostic substitutions (community 170 and hs_SVA_D6d-f in community 1).

The first subfamilies to branch off in the diagnostic substitution-based network (Fig. 3a) are characterized by one (pt_DRd/hs_D4/pt_D6) and two (hs_D8 and gg_D3a → hs_D7) shared diagnostic substitutions, respectively (see also yellow bars in Fig. 3d). In Levy et al.’s network the corresponding communities (230 → 172 and 170) appear as descendants of community 2 whose constituents are characterized by two additional shared diagnostic substitution (Fig. 3b, d). Considering that diagnostic substitutions representing mutations inherited from a source element are acquired in a sequential manner, community 2 cannot be the ancestor of the two communities in question.

Further on, analysis of diagnostic substitutions provides little support for pt_D4 (D3_Pt, community 36; Fig. 3b, d) elements being derived from the node community 2 as such a path would involve back-mutation of an “A” to “G”. The same applies for the related human subfamilies hs_D6d-f present in community 1 (green bar). A separate human-chimpanzee network covering human hs_D6 and the related chimpanzee subfamilies (Fig. 3c) provides support for two clearly distinguished lineages: the “AG” lineage including subfamilies hs_D6b/6c and pt_D5a-c and the “GT” lineage comprising hs_D6d-f and chimpanzee pt_D4a-c. Last but not least the hs_D6a elements (three diagnostic substitutions) cannot be derived from elements present in community 2.

Comparison of communities 1 and 2 (Fig. 3b, d) revealed that all subfamilies present in the “ancestral” community 2 are also represented in the “derived” community 1. Split of subfamilies (defined by diagnostic substitutions reflecting common origin) between communities is also observed in the species-specific networks presented by Levy et al. [10]. In chimpanzee communities D1 and D1b both contain members of the same six subfamilies. In the human network obtained at a higher resolution (θ 0.9) two subfamilies are split between communities D1b and D1d. Another case is found in the SVA_C part of the all-hominid network where a clearly distinguishable sub-group in C is split between the communities presented in the network (for details see Additional file 4: Note S1, Tables S2, S3; Figure S1).

In the lower resolution per-species networks (θ = 0.8 and 0.9; [10]) two of the subfamilies re-analyzed are not clearly delimited: human SVA_D5 (θ = 0.8) contains a substantial fraction of elements (18 out of 84; 21%) lacking the SVA_D5 diagnostic residues; human SVA_D1d (θ = 0.9) consists of 55% elements of hs_D6a-f, 9% elements belonging to four different smaller subfamilies and 36% SVA_DR elements (for details see Additional file 4: Note S1, Table S3). Concluding from the finding that both SVA_D5 and SVA_D1d are clearly delimited at higher resolution (θ = 0.9 and θ = 0.92, respectively) it has to be assumed that elements not belonging to the core constituent subfamilies SVA_D5 and SVA_D1d (hs_D6a-f) are “back-sorted” to more ancestral subfamilies (D1b, c).

Neither split of subfamilies containing elements of common descent nor “sorting back” of elements to a more ancestral subfamily at higher resolution occurs in diagnostic substitution-based subfamily establishment.

Finally, the phylogenetic relationships of gorilla SVA_D elements as established by Levy et al. [10] do not differ from those found based on diagnostic substitutions – with gg_D4 giving rise to gg_D5a-c (D3_Gg) on the one hand and to gg_D4b-h (D4_Gg) on the other hand. However, the intermediate node leading to D4_Gg (Fig. 3b, community 21) is a collection combining elements of several D4 subgroups that lack one or two of the subgroup-specific diagnostic residues with elements that cannot safely be assigned to any of the D4 subgroups due to 5′ truncation and two elements clearly belonging to the distal and most divergent gg_D4d subfamily.

### Tracing the origin of the human-specific SVA_F subfamily

**Fig. 4:**
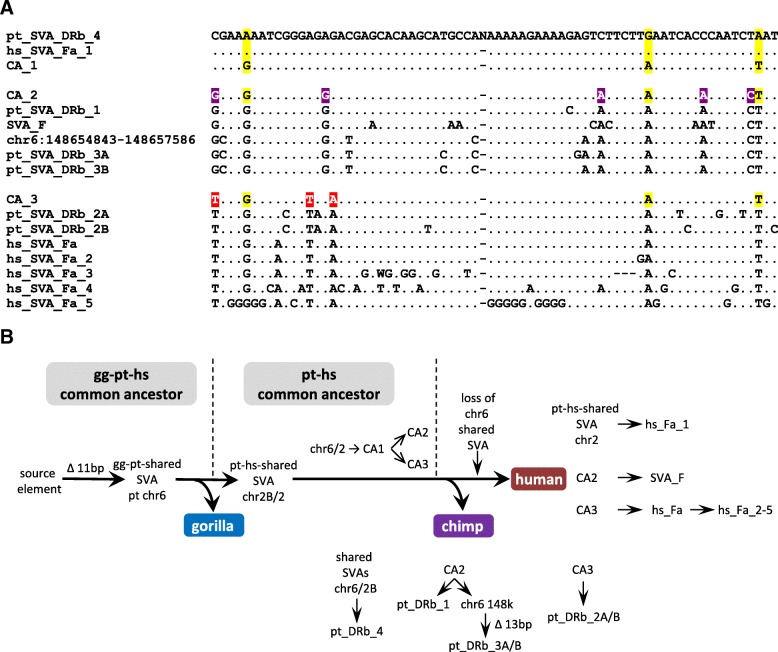
The origin of the human-specific subfamily SVA_F. **a** Alignment of the consensus sequences of pt_SVA_DRb and hs_SVA_Fa sub-groups and SVA_F. Only variant positions are shown. The chromosome 6 element included represents the source element of pt_SVA_DRb_3 still lacking a sub-group-specific additional 13 bp deletion (not included in the alignment) in the *Alu*-like region. CA_1–3 represent the inferred sequences of the common ancestors of the entire cluster (CA_1); the pt_SVA_DRb_1/pt_SVA_DRb_3/SVA_F group (CA_2, top panel) and the pt_SVA_DRb_2/hs_SVA_Fa group (CA_3, bottom panel). pt_SVA_DRb_4 and hs_SVA_Fa_1 are the sub-groups containing the elements shared between gorilla/chimpanzee and chimpanzee/human, respectively. Substitutions leading to CA_1 are highlighted in yellow; those leading from CA_1 to CA_2 in purple and those resulting in CA_3 in red. N represents the junction between the *Alu*-like and SINE-R domains. Substitutions in the chr6 element not present in any of the consensus sequences were assumed to represent random mutation and disregarded. Some of the pt_SVA_DRb_4 elements in chimpanzee represent an additional G → C mutation absent from the gg-pt shared chr6 element and the pt-hs shared chr2 element in humans – it was, therefore, considered to represent a chimpanzee-specific mutation and excluded from the pt_SVA_DRb_4 consensus. **b** A scenario for the emergence of the human-specific SVA_F and related subfamilies. The 11 bp deletion in the SVA *Alu*-like domain to become the hallmark of human SVA_F elements occurred in the hominine common ancestor. The resulting gg-pt-shared element on chromosome 6 gave then rise to the pt-hs-shared chr2 element in the chimpanzee-human common ancestor. Either one of the two shared elements currently present in chimpanzee could have been the precursor of a common ancestor (CA_1) that subsequently gave rise to two distinct lineages founded by CA_2 and CA_3. After the chimpanzee-human split species-specific amplification starting from these two founders and the shared elements occurred in both humans and chimpanzees. The gg-pt-shared chr6 element has been lost in the human lineage. gg – gorilla; pt – chimpanzee; hs – human; CA – common ancestor; chr6 148 k – pt_DRb3 precursor lacking the 13 bp additional deletion

**Fig. 5:**
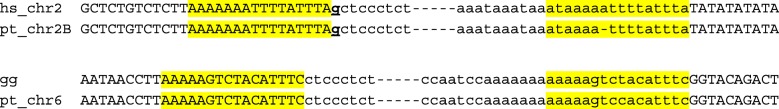
Inter-species alignments of the shared potential SVA_F source elements. Only the 5′ and 3′ ends (separated by spacer) of the SVA elements are shown. Target site duplications are highlighted in yellow. The “g” highlighted (bold, underlined) in the chromosome 2/2B element is untemplated and can be attributed to capping of the SVA RNA. gg – gorilla; pt – chimpanzee; hs – human

In their all-hominid network Levy et al. [10] observed chimpanzee SVA elements in the “previously thought to be human-specific” subfamily SVA_F. They suggest that this could be attributed to either “the initial proliferation of an element that was present in a common ancestor or due to convergent evolution”. Identification of shared elements in the community pointed towards a source element in the hominine or human-chimpanzee common ancestors. Intrigued by this finding I set out to characterize the involved chimpanzee and human SVA communities in more detail. Analysis of the community comprising human SVA_F and chimpanzee elements revealed that it contains a distinct group of both human and chimpanzee elements that phylogenetic analysis using the SINE-R part placed in between subfamilies hs_SVA_D5 and SVA_F. The human elements of this group had been identified as hs_SVA_Fa (Fig. 2) and by Levy et al. as SVA_F_1 (human network at θ = 0.9 [10]). The chimpanzee elements fell into subfamily pt_SVA_DRb (Fig. 2). Both hs_SVA_Fa and pt_SVA_DRb already present the 11 bp SVA_F hallmark deletion in the *Alu*-like region. Closer inspection revealed the presence of sub-groups within both subfamilies (pt_SVA_DRb_1–4 and hs_SVA_Fa/Fa_1–5; Fig. 4a). Comparative genomics identified two elements shared between species: one on human chromosome 2 with chimpanzee (hg19 chr2:203,398,957-203,400,849 and panTro5 chr2B:93,201,203-93,203,235), the other one on chimpanzee chromosome 6 is shared with gorilla (panTro4 chr6:145,572,534-145,575,253 and gorGor5 CYUI01014937v1:13,195,711-13,197,203). Alignments covering the flanking regions and TSDs of the elements are given in Fig. 5. These SVAs belong to sub-groups pt_SVA_DRb4 (5 elements in total) and hs_SVA_Fa_1 (3 elements in total). From the point of view of evolution the existence of a gorilla-chimpanzee shared element displaying the 11 bp deletion in the *Alu*-like region places the origin of the entire “cluster” in the gorilla-chimpanzee-human common ancestor (Fig. 4b). Following split-off of gorilla the chr6 element gave rise to the chimpanzee-human-shared chr2 element and either one of the elements gave rise to an ancestral element (CA_1; Fig. 4a and b) characterized by three substitutions. Further on two different lineages evolved (top and bottom parts of the alignment in Fig. 4a); founded by CA_2 and CA_3, respectively. Split into these two lineages must have occurred in the chimpanzee-human common ancestor because both of them contain chimpanzee and human sub-groups. Following separation of chimpanzees and humans

- the shared elements gave rise to pt_SVA_DRb4 and hs_SVA_Fa_1, respectively
- CA_2 gave rise to pt_SVA_DRb1 and pt_SVA_DRb3 in chimpanzee and SVA_F in humans
- CA_3 gave rise to pt_SVA_DRb2 in chimpanzee and hs_SVA_Fa in humans.

This scenario represents the most parsimonious explanation for the evolution of chimpanzee DRb and human SVA_Fa/SVA_F elements – taking into account the two shared SVAs and available sequence information. It also suggests that SVA_D5 (a separate subfamily in humans and a small subgroup in chimpanzees; see Fig. 2) as it exists today is not the “precursor” of the elements containing the 11 bp deletion in the *Alu*-like region – rather it evolved independently. This notion is also supported by the fact that in all networks rooted on SVA_D5 the sub-groups containing the shared elements are found in the distal, most divergent part of the networks (not shown).

### Differences in SVA lineage-specific expansion in gorilla/chimpanzee versus human

Scrutinizing the SINE-R consensus sequences obtained I noticed that in quite a number of subfamilies the SINE-R is characterized by hallmark A-G co-segregating substitutions at positions 228 and 242 relative to the SVA_DR consensus (Additional file 5). Following up on this observation I determined that 59% and 53%, respectively, of the lineage-specific insertions in gorilla and chimpanzee share these substitutions (Additional files 1 and 5, Fig. 2). In humans only 10% of the lineage-specific SVA_D elements fall into this category. By contrast, in humans the major expanding SVA subfamily is SVA_F, canonical SVA_F elements constituting around 23% of all human lineage specific insertions. Why there are differences in the major expanding subfamilies between humans (SVA_F) versus chimpanzees (“A-G” subfamilies) although precursor of the “A-G” subfamilies expanding in chimpanzee exist in humans as well (hs_SVA_D6b) cannot be explained based on current knowledge. Possibly, the *Alu*-like domain containing the 11 bp deletion represents an advantage in the human environment – either by being a better substrate for human L1 elements or in interaction with host factors.

### The existence of an SVA “master” element active over extended periods in evolution is not very likely

**Fig. 6:**
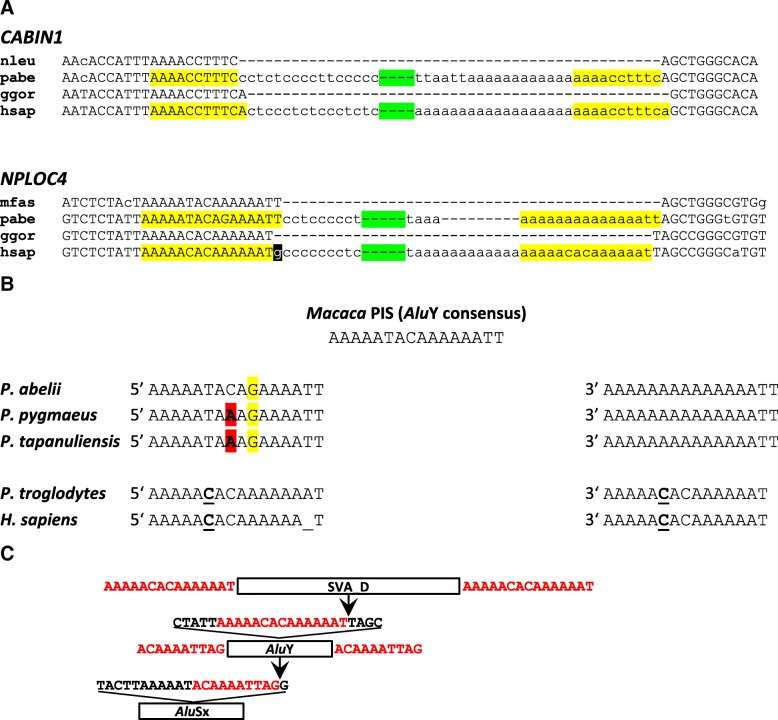
SVA elements in the *NPLOC4* and *CABIN1* genes. **a** Inter-species alignments of the pre-integration and SVA-containing loci in the *NPLOC4* and *CABIN1* genes. Only the 5′ and 3′ ends (separated by a green spacer) of the SVA elements are shown. Target site duplications (TSDs) are highlighted in yellow. The “g” highlighted in the human element is untemplated and can be attributed to capping of the SVA RNA. nleu – *Nomascus leucogenys*; pabe – *Pongo abelii*; ggor – *Gorilla gorilla*; hsap – *Homo sapiens*; mfas – *Macaca fascicularis* (**b**) Comparison of the TSDs of the elements integrated into the *NPLOC4* gene to the inferred pre-integration site (PIS) and across species. 5′ and 3′ indicate the 5′ and 3’ TSDs, respectively. Mutations in the orangutan TSDs are highlighted in yellow and red. The substitution distinguishing the human/chimpanzee TSDs from the PIS is underlined. **c** Integration of SVAs into the *NPLOC4* locus occurred close to an *Alu* internal A tract. In the course of evolution insertion of an *Alu*Sx was followed by retrotransposition of an *Alu*Y directly downstream of the *Alu*Sx internal A tract. A position one nucleotide downstream of the *Alu*Y internal A tract was then targeted by the SVA insertion. Only the SVA_D insertion present in humans and chimpanzees is shown. Target sites and target site duplications are shown in red

Based on the analysis of orangutan-human orthologs the recent publication by Levy et al. suggests the existence of a “master RE [retroelement] that was active over a long period in evolution, spawning subfamilies A to D subsequently, as it accumulated mutations over time” [10]. In support for the hypothesis two loci containing SVA_A in orangutan and SVA_D in humans and chimpanzees are presented. According to the analyses supplied in the supplement the authors claim that “it is very unlikely that the *CABIN1* or *NPLOC4* elements represent independent insertions in the orangutan and human-chimp genomes”. Re-analysis, however, provides evidence that the SVA_D (human/chimpanzee) elements in the *NPLOC4* and *CABIN1* genes have been inserted very close to but not into the exact same integration site as the orangutan SVA_A elements. Fig. 6a shows inter-species alignments of the pre-integration sites and the filled alleles in orangutan and humans. The integration sites between orangutan and human differ by one nucleotide in both cases.

In case of the SVAs present in the *NPLOC4* locus analysis of the target site and TSDs in different species provides further support for an independent integration event in the human/chimpanzee ancestor (Fig. 6b; for details on the identification of supporting short reads see Additional file 4: Note S2). The ancestral state of the target site in an *Alu*Y can be inferred from its sequence in Old World monkeys (*Macaca*; gibbon sequence for the locus is not available); it corresponds to the *Alu*Y consensus (AAAAATACAAAAAATT). In orangutan two alleles can be found for the 5’TSD: AAAAATACA**G**AAAATT (*P. abelii* except for Dunja whose taxonomic status is unclear) and AAAAATA*A*A**G**AAAATT (*P. pygmaeus* and *P. tapanuliensis*). The A → G mutation at position 10 must have occurred in a common orangutan ancestor as it is found in all three species. The fact that the C → A mutation at position 8 is shared between *P. tapanuliensis* and *P. pygmaeus* can probably be explained by differential sorting of the two possible alleles (C/A) at the split of *P. tapanuliensis* and *P. abelii*. *P. tapanuliensis* is more closely related to *P. abelii* than to *P. pygmaeus* [14]. The 3’ TSD, AAAAAAAAAAAAAATT in all three species, is characterized by two mutations compared to the inferred ancestral state (T → A at pos. 6 and C → A at pos. 8). Independent mutations occurring in 5′ and 3’ TSDs are to be expected considering the age of the SVA element inserted. In humans, by contrast, 5′ and 3’ TSD are completely identical (AAAAA**C**ACAAAAAAT – one mutation compared to the inferred ancestral state). In chimpanzees the 5’ TSD contains an additional “A” that can be attributed to replication slippage.

If indeed the *NPLOC4* SVA_D in the human/chimp lineage evolved from the SVA_A present in orangutan then, based on age, independent mutations in the 5′ and 3’ TSDs would be expected in humans/chimpanzees, too – similar to the divergent TSDs in orangutan. The exactly same mutations independently occurring in both TSDs is not very likely. Rather, it occurred in the target site in the human/chimpanzee common ancestor. SVA_D insertion then generated two completely identical TSDs as they are visible in humans. In addition the 5′ untemplated G, attributed to capping of the SVA RNA is discernible in case of the SVA_D in human/chimpanzee but not found in the orangutan SVA_A (Fig. 6a). Finally, my own analysis did not provide any evidence for the presence of an SVA in the *NPLOC4* locus in gorilla (for details see Additional file 4: Note S2, Figure S2, Table S4). Absence in gorilla points to an independent SVA_D integration event in the chimpanzee-human common ancestor after the split-off of gorilla.

Neither in case of the elements in the *CABIN1* gene did my own analysis provide support for the presence of the SVA in gorilla. Extensive Blast analysis identified a single read pair with one arm matching the 3′ flanking sequence and the other an SVA (also identified by Levy et al. [10]). However, both reads in the pair correspond in sequence to orangutan. Possibly the pair represents a contamination – gorilla and orangutan genomic DNAs were sequenced at the same center. For the second read pair identified by Levy et al. for the *CABIN1* SVA the authors provided the sequence of the SVA-matching upstream read and the name of the individual it was derived from (Kolo). Blast search against the Kolo SRAs (short read archives) retrieved four read pairs with the upstream read 100% identical to the sequence provided over the entire length of 100 bp. None of the corresponding downstream reads, which all overlap the SVA polyA tail, can be mapped to the *CABIN1* locus (alignments are provided in Additional file 4: Figure S3).

Integration of the SVAs into the *NPLOC4* locus occurred downstream of an *Alu*Y internal A tract (Fig. 6c). The integration site is localized one (human/chimpanzee) respectively two (orangutan) nucleotides downstream of a hotspot described for insertion of *Alu*s into *Alu*s (directly adjacent to the *Alu* internal A tract [15]). The *NPLOC4* locus had been the target of transposable element insertion before: the *Alu*Y now interrupted by the SVAs has itself integrated into an *Alu*Sx that jumped into the locus first. In this case, too, integration occurred at a short distance downstream of the internal A tract (Fig. 6c).

The elements found in the *CABIN1* locus inserted directly adjacent to an *Alu*Sq2 element. An insertion preference of SVAs into or close to *Alu* TSDs has been reported [3].

In addition to the findings concerning the integration events, it has to be taken into account that evolution of SVA elements does not only involve nucleotide exchanges and the appearance of indels in the *Alu*-like and SINE-R domains, but also the emergence of a highly ordered VNTR structure with subfamily-specific subunits [9]. It is difficult to conceive for all of these to have occurred in a single element.

### Species-specific non-canonical SVA subfamilies – A striking case of SVA parallel evolution in chimpanzees and humans

**Fig. 7:**
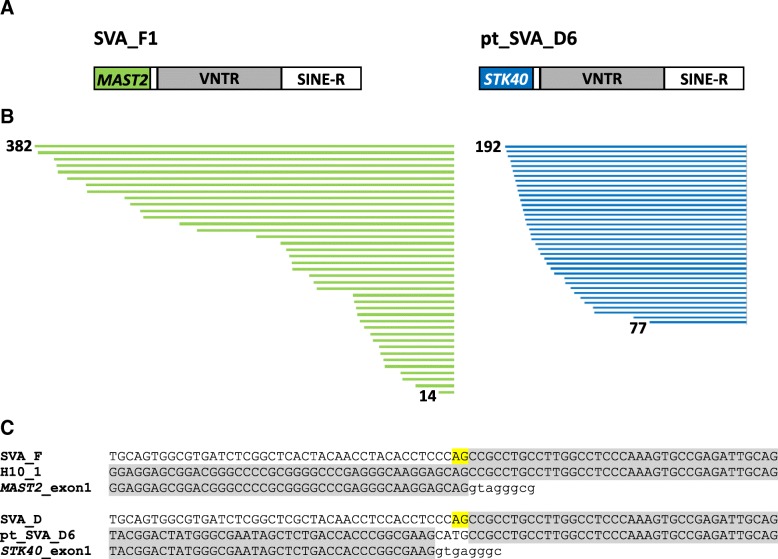
Parallel evolution of SVA structural variants in humans and chimpanzees. **a** Schematic representation of SVA_F1 (human) and pt_SVA_D6 (chimpanzee) elements. In both cases the 5’ *Alu*-like region is replaced by the GC-rich first exon of a gene encoding a serine-threonine kinase: *MAST2* in SVA_F1 and *STK40* in pt_SVA_D6. **b** Length distribution of the acquired exon sequences for SVA_F1 and pt_SVA_D6. Identical lengths of *MAST2*/*STK40* acquired sequences in multiple elements are represented only once. The data for SVA_F1 were taken from Damert et al. [6]. **c** Multiple alignments of canonical SVA (*Alu*-like) sequences (SVA_D and SVA_F), the first exons of *MAST2* and *STK40*, and the resulting SVA_F1 and pt_SVA_D6 elements. The splice acceptor “AG” is highlighted in yellow. H10_1: SVA_F1 source element [6]

Scrutiny of SVAs in chimpanzees revealed that in subfamily pt_SVA_D6 the 5′ hexameric repeats and the larger part of the *Alu*-like domain have been replaced by non-SVA sequence. Further analysis identified this sequence as the first exon of the *STK40* gene. Chimpanzee pt_SVA_D6 can, therefore, be considered the counterpart to the human SVA_F1 subfamily in which the first exon of the *MAST2* gene has been spliced to an acceptor at the 3′ end of the *Alu*-like domain [6–8] (Fig. 7). Based on the fact that the *STK40*-derived sequence coincides with the exon 1 3′ end, splicing seems the most likely mode of its acquisition, even if the SVA splice acceptor cannot be reconstructed by comparison to the SVA_D consensus (Fig. 7c). If it had not been present in an extinct pt_SVA_D6 source element, the CATG tetranucleotide could be the remainder of an earlier 5′ transduction which in turn provided the splice acceptor for the acquisition of the *STK40* exon. Pt_SVA_D6 comprises 94 elements (panTro4/supplemented with panTro5) that contain the *STK40* sequence. Many of them contain additional 5′ transductions – among them exons of 8 different other genes (Additional file 6). Interestingly, the subfamily also comprises three canonical SVA elements. However, none of them contains the CATG tetranucleotide.

The similarities between human SVA_F1 and chimpanzee pt_SVA_D6 are striking: in both cases (i) first exons of genes encoding serine-threonine kinases have been co-opted, (ii) the exons are GC-rich and can serve as internal promoters (experimentally proven for SVA_F1 [16], to be assumed for pt_SVA_D6) and (iii) the source genes, *STK40* and *MAST2*, have their highest expression in testis (according to the RNAseq data provided in the NCBI Gene section).

Considering that both pt_SVA_D6 (94) and SVA_F1 (84 [6]) reached relatively high copy numbers when compared to other 5′ transduction groups containing parts of spliced mRNAs [6], one could speculate that replacement of the hexameric repeat/*Alu*-like region by a GC-rich exon confers an advantage with regard to either expression (as a pre-requisite of mobilization) or with regard to mobilization itself. SVA_F1 and pt_SVA_D6 may, thus, represent the “evolutionary future” of this family of composite non-LTR retrotransposons.

## Methods

### Sequence retrieval, alignment and sorting into subfamilies

The SINE-R domains of chimpanzee (panTro3) and human (hg19) SVA_D elements were extracted using the coordinates provided by Levy et al. [10] and the UCSC genome browser table browser tool. In case of chimpanzee the panTro3 coordinates were converted to panTro4 before extraction. For gorilla all SVA elements repeat-masked SVA_D were extracted from gorGor5. The choice of genome build for each of the species has been dictated by their quality and the availability of data from previous analysis: compared to the latest build (hg38) human hg19 does not represent significant differences with regard to SVAs; for chimpanzee part of the data had already been available from an earlier analysis based on panTro4 [9], a cursory analysis of panTro5 revealed that it does not present major advantages over panTro4 as far as gaps in and misassembly of SVAs is concerned. In case of gorilla gorGor5 is the first genome build permitting a rigorous genome-wide analysis of SVA elements. Entire SVA elements (gorilla) or the SINE-R parts (chimpanzee, human) were aligned in BioEdit and manually sorted into subfamilies based on shared substitutions distinguishing them from a reference (SVA_C and SVA_D [1]). A minimum of two substitutions shared among the members of a subfamily and distinguishing them from the reference was used as sorting criterion. Groups with a minimum number of 10 elements were considered to represent a subfamily; with exception of hs_SVA_D7 and pt_SVA_DRc which have equivalents in other species and the well-defined hs_SVA_DRc. Elements that could not be properly aligned or lacked more than half of the SINE-R domain were excluded from the analysis. Consensus sequences were generated using a majority rule. Consensus sequences of SVA_C, SVA_D, SVA_E and SVA_F were those reported by Wang et al. [1]. Sequences of the *Alu*-like domains of chimpanzee pt_D5 and pt_DRb and human hs_D5 and hs_Fa elements were retrieved manually using the UCSC genome browser.

### Determination of species-specificity of SVA insertions

Species-specificity of integration events was determined using LiftOver between the above mentioned genome builds. Only in case of the gorilla – human comparison LiftOver was effected between gorGor5 and hg38 as no direct LiftOver to hg19 is available. In case of human and chimpanzee the positions of the SINE-R domains were used; for gorilla the positions of the SVA_D-repeat-masked elements. All elements indicated to be shared by LiftOver were manually inspected using the UCSC genome browser alignment nets to ensure correct conversion. Where necessary, panTro4 positions were converted to panTro5 – which in some cases provided gap-free alignment nets.

### Phylogenetic analysis

Median-joining networks were constructed using Network 4 [17] with default settings. The MP option for post-processing [18] was used for all initial networks.

### Analysis of SVA communities identified by Levy et al. [10]

The dataset of subfamily-assigned SVAs provided in the supplement [10] was filtered for the respective subfamily or community. Communities in the all-hominid network were identified based on the number of community members and the subfamily affiliation in the lower resolution networks. The positions of the SINE-R domains were extracted and the corresponding sequences retrieved from gorGor3, panTro3 and hg19, respectively, using the UCSC table browser tool. Sequences were aligned in BioEdit and manually sorted into the SVA subfamilies established by diagnostic residue-based sorting.

## Additional files

Additional file 1: SVA_D elements in gorilla, chimpanzee and humans. Subfamily denominations, total number of elements in the respective subfamily and numbers of elements shared between lineages are provided for gorilla, chimpanzee and humans. (XLSX 16 kb)

Additional file 2: Genomic coordinates, subfamily affiliation and species-specificity of SVA_D elements in hominine primates. (XLSX 182 kb)

Additional file 3: Consensus sequences of the SINE-R domains of SVA_D subfamilies in gorilla (gg), chimpanzee (pt) and humans (hs). (TXT 31 kb)

Additional file 4: Supplementary tables, notes and figures. (PDF 518 kb)

Additional file 5: Multiple alignment of the consensus sequences of SVA_D subfamilies containing the hallmark A-G co-segregating substitutions at positions 228 and 242 relative to the SVA_DR consensus. (PDF 72 kb)

Additional file 6: pt_SVA_D6 – a subfamily of non-canonical SVA elements in chimpanzees. Positions, target site duplications, length of the *STK40*-derived sequence and details on additional 5′ transductions are provided. (XLSX 21 kb)

## Abbreviations

LINE: Long Interspersed Element
LTR: Long Terminal Repeat
myrs: Million years
RE: Retroelement
SINE: Short Interspersed Element
SINE-R: SINE of retroviral origin
SRA: Short read archive
SVA: SINE-R-VNTR-*Alu*
TSD: Target Site Duplication
VNTR: Variable Number of Tandem Repeats
