# Supplementary material for: Phylogenomic analysis reveals splicing as a mechanism of parallel evolution of non-canonical SVAs in hominine primates
Source: Mob DNA. 2018 Sep 17;9:30. doi: 10.1186/s13100-018-0135-2 (PMC6139936; doi:10.1186/s13100-018-0135-2)
Supplement: Supplementary file 4 — Supplementary tables, notes and figures. (PDF 518 kb) [file 13100_2018_135_MOESM4_ESM.pdf]

Additional file 4 Supplementary tables, notes and figures

Table of contents

- Table S1 Subfamilies recovered in this analysis and by Levy et al. [1]
- Note S1 Split of subfamilies between communities
- Table S2 Composition of chimpanzee communities D1 and D1b (θ 0.8)
- Table S3 Composition of human communities D1b, D1c and D1d (θ 0.9)
- Figure S1 Consensus sequence of the SINE-R of the sub-group of SVA_C elements split between communities 99 and 139.
- Note S2 Analysis of gorilla and orangutan short read archives to detect the SVA in the *NPLOC4* locus
- Figure S2 The SVA_D element in the human *NPLOC4* locus is surrounded by interspersed repeats.
- Table S4 Summary statistics of the "anchored" Blast search for the *NPLOC4* element in gorilla
- Figure S3 Multiple alignment of read pairs retrieved from gorilla "Kolo" short read archives
- Additional References

Table S1 Subfamilies recovered in this analysis and by Levy et al. [1]

Species	subfamily	subfamily Levy et al. [1]	observations
G. gorilla	gg_SVA_DR	SVA_D1_Gg (+113G)	
	gg_SVA_D2a	SVA_D2_Gg	
	gg_SVA_D5b	SVA_D3_Gg	
	gg_SVA_D4c	SVA_D4_Gg	
P. troglodytes	pt_SVA_DR	SVA_D1_Pt/ SVA_D1b_Pt*	
	pt_SVA_D2b	SVA_D2_Pt	
	pt_SVA_D4a	SVA_D3_Pt	
	pt_SVA_D6	SVA_D4_Pt	
	pt_SVA_D5b	SVA_D5_Pt	
H. sapiens	hs_SVA_DR	SVA_D1	
	hs_SVA_D2a	SVA_D2	
	hs_SVA_D3	SVA_D3	
	hs_SVA_D4	SVA_D4	
	hs_SVA_D5	SVA_D5	
	hs_SVA_DRb	SVA_D6	
G. gorilla	gg_SVA_D1	D1a	all-hominid network
	gg_SVA_DRb	(SVA_E)	as part of E in all-hominid network
	gg_SVA_DRc	D6	as part of D6 in all-hominid network
P. troglodytes	pt_SVA_D1	D1a	all-hominid network
	pt_SVA_DRd	part of D4	all-hominid network
	pt_SVA_D3	D3	all-hominid network
H. sapiens	hs_SVA_D1	D1a	Θ 0.9
	hs_SVA_D4	D4	all-hominid network
	hs_SVA_D6a-f	D1d	recovered at Θ 0.9 as part of D1d; in all-hominid network as separate SF in D1d/Pt_SVA_D5; no split into constituent SFs
	hs_SVA_D7	D1d	combined in 69-member-D1d community 170 in all-hominid network
	hs_SVA_D8	D1d	
	hs_SVA_Fa	SVA_F_1	Θ 0.9; not recovered in all-hominid network

The upper part of the table lists subfamilies for which consensus sequences are provided by Levy et al. [1]; the lower part lists subfamilies for which identity was established by analysis of the data provided in the supplementary information to Levy et al. [1]. SF – subfamily; * subfamilies with identical consensus sequences

Note S1 Split of subfamilies between communities

Communities identified by the network-based approach [1] were re-analyzed by retrieving the sequences of the elements based on the positions provided in the supplementary material, aligning them and sorting them into the subfamilies identified by diagnostic substitution-based sorting. Chimpanzee communities D1 and D1b (θ 0.8; Fig. 2 in [1]) both contain members of subfamilies pt_D1, pt_DRa-d and pt_D3 (Table S2).

Table S2 Composition of chimpanzee communities D1 and D1b (θ 0.8)

SF (Levy) \ SF	Total nr	pt_SVA_ D1	pt_SVA_ DRa	pt_SVA_ DRb	pt_SVA_ DRc	pt_SVA_ DRd	pt_SVA_ D3	pt_SVA_ D5a
**D1_Pt**	328	66	10	27	5	4	15	8
**D1b_Pt**	243	45	9	40	5	5	16	

Table S3 shows the composition of human communities D1b, D1c and D1d identified by Levy et al. at higher resolution (θ 0.9 [1]).

Table S3 Composition of human communities D1b, D1c and D1d (θ 0.9)

SF (Levy) \ SF	Total nr	hs_ DRa	hs_ D7	hs_ D8	hs_ DRc	hs_ D6a	hs_ D6b	hs_ D6c	hs_ D6d	hs_ D6e	hs_ D6f	hs_SVA D5/D5b
**SVA_D1b**	65	13	1	1	2							1D5
**SVA_D1c**	242	21			2							1D5b
**SVA_D1d**	353	5	7	18	3	26	73	24	27	32	11	

The last example of subfamilies split between communities is found in the all-hominid network where a clearly defined sub-group in C is split between communities 99 and 139. Figure S1 shows the consensus sequence for this subgroup in comparison to SVA_C. 31 members of the sub-group are found in community 99 and 32 in community 139.

Figure S1 Consensus sequence of the SINE-R of the sub-group of SVA_C elements split between communities 99 and 139. Variant residues are highlighted in yellow.

```
SVA_C      CAGCTCATTGAGAACGGGCCATGATGACGATGGCGGTTTTGTCGAATAGAAAAGGGGGAAATGTGGGGAAAAGATAGAGA
C_variant  ..........................................G.....................................

SVA_C      AATCAGATTGTTGCTGTGTCTGTGTAGAAAGAAGTAGACATAGGAGACTCCATTTTGTTCTGTACTAAGAAAAATTCTTC
C_variant  ........................................................................G.......

SVA_C      TGCCTTGGGATGCTGTTGATCTATGACCTTACCCCCAACCCGGTGCTCTCTGAAACATGTGCTGTGTCCACTCAGGGTTA
C_variant  ..............................G..........T......................................

SVA_C      AATGGATTAAGGGCGGTGCAAGATGTGCTTTGTTAAACAGATGCTTGAAGGCAGCATGCTCGTTAAGAGTCATCACCACT
C_variant  ................................................................................

SVA_C      CCCTAATCTCAAGTACCCAGGGACACAAACACTGCGGAAGGCCGCAGGGTCCTCTGCCTAGGAAAACCAGAGACCTTTGT
C_variant  ................................................................................

SVA_C      TCACTTGTTTATCTGCTGACCTTCCCTCCACTATTGTCCTATGACCCTGCCAAATCCCCCTCTGCGAGAAACACCCAAGA
C_variant  ................................................................................

SVA_C      ATGAT
C_variant  .....
```

Note S2 **Analysis of gorilla and orangutan short read archives to detect the SVA in the *NPLOC4* locus**

Levy et al. [1] identified 13 SVA 3’ (SINE-R) read pairs with “one arm mapping uniquely to the flanking regions and the other to an SVA element” from gorilla short read archives by mapping the datasets to the human sequence flanking the SVA insertion. The analysis of the locus using short read archives is complicated by the fact that the target site is completely surrounded by repetitive sequence (mostly *Alu*): 42% of the sequence flanking the SVA at the 5’ end are repeat-masked; the 1kb downstream completely consist of interspersed repeats – except for the 3’ most section (Fig. S2). Human and gorilla sequences in the region 1kb downstream of the SVA insertion site are 98% identical (excluding gaps). If only the relevant 600bp (if one arm of the read should match the SVA and the other one the flanking region then, considering fragment lengths in the libraries, the 3’ read can map at maximum 600bp downstream) are taken into account, identity between human and gorilla is 97%. In Blast searches performed to detect the SVA in the gorilla *NPLOC4* locus hits with less than 98% identity to the query were in general found to match to other *Alu*s in the genome by BLAT. Thus, a read with a 100% match to the human sequence is likely to map to a different position in the gorilla genome.

To avoid false-positives resulting from retrieval of reads mapping to highly similar *Alu* sequences elsewhere in the genome I took advantage of the fact that approximately 400bp upstream of the integration site there is a stretch of non-repetitive sequence. I used this stretch to “anchor” a Blast search by using a query overlapping the non-repetitive sequence and the 5’ end of the *Alu*Jb. Provided that fragment length in the libraries is >350bp this approach should recover read pairs with the 5’ read “anchored” in non-repetitive sequence and the 3’ read localizing to the SVA – if it is present.

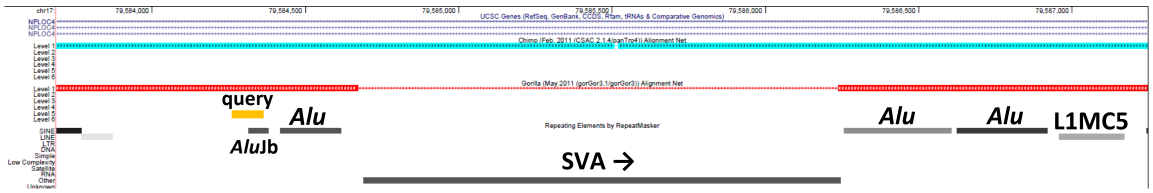

Figure S2 **The SVA_D element in the human *NPLOC4* locus is surrounded by interspersed repeats.**
The SVA element and 1kb each of up- and downstream flanking sequence is shown. Comparison of the chimpanzee (light blue) and gorilla (red) alignment nets indicates the absence of the SVA in the gorilla reference genome build. The position of the 5’ Blast query “anchored” in non-repetitive sequence is indicated by an orange bar.

Paired-end short read archives are available for 26 gorilla individuals. For 18 of these individuals the average library fragment length is larger than 350bps. However, in none of the datasets a 3’ read covering SVA sequence could be identified, although the insertion site is covered: reads overlapping the empty

target site were recovered for all individuals; in some cases also 3’ reads mapping downstream of it could be identified – which, given library fragment size, would be impossible if the SVA is present. In parallel short read archives of 11 orangutans were analyzed using the same approach. In all of them all 3’ reads extending past the TSD contained SVA sequence matching that of the orangutan SVA_A (Table S4). No 3’ reads corresponding to the empty pre-integration site were recovered. For the remaining 8 gorilla individuals with library fragment length smaller 350bp Blast search was performed using a query spanning 100bp each up- and downstream of the target site. Mapping of the read pairs to the *NPLOC4* locus was verified using BLAT. Blast hits below 98% identity to the query were found to match to other loci in the genome and discarded. For the 8 individuals between 5 and 17 reads confirming the absence of the SVA (i.e. matching the empty pre-integration site) were identified. No evidence was found for an SVA inserted into the target site. However, this approach is more likely not to detect the SVA-containing allele in a Blast search as longer hits with less similarity matching other *Alu* sequences in the genome might outnumber shorter hits matching only part of the query (the other part being SVA sequence) with higher similarity at a given maximum number of hits to return. Consequently, the shorter hits might not be reported.

Table S4 Summary statistics of the "anchored" Blast search for the *NPLOC4* element in gorilla

individual	total nr of read pairs	3' read 5' of TSD	3' read over-lapping TSD	3' read 3' of TSD	average fragment length*
CAROLYN	6	1	2	3	475
DIAN	13	0	5	8	524
MIMI	9	2	7	0	451
SANDRA	10	0	8	2	487
KATIE	11	3	7	1	426
DOLLY	3	0	3	0	464
OKO	10	8	2	0	376
HELEN	5	2	1	2	422
VILA	4	0	3	1	480
PORTA	6	4	1	1	388
ANTHAL	11	3	7	1	416
PAKI	8	2	6	0	436
CHOOMBA	9	3	3	3	478
AMANI	11	0	7	4	500
KOLO	7	0	4	3	485
COCO	15	0	11	4	473
DELPHI	17	3	4	10	483
BANJO	9	1	6	2	485
SUZIE	10	9	1	0	319
AZIZI	10	9	1	0	270
BULERA	9	8	1	0	289
AKIBA	1	1	0	0	411
TZAMBO	4	4	0	0	210
ABE	2	2	0	0	186
KOKOMO	10	10	0	0	264
KOWALI	10	10	0	0	284
**individual**	**total nr of read pairs**	**3' read 5' of TSD**	**3' read over-lapping TSD**	**3' read in SVA**	**average fragment length**
			P. abelii		
ELSI	3	0	3	0	481
KIKI	1	0	0	1	518
DUNJA	10	4	6	0	454
BABU	4	1	3	0	450
BUSCHI	6	4	2	0	445
			P. pygmaeus		
NAPOLEON	9	2	6	1	462
NONJA	5	2	2	1	476
SARI	4	0	2	2	493
TEMMY	5	1	2	2	405
TILDA	2	1	1	0	447
P. tapanuliensis	31	28	2	1	326

* calculated based on the read pairs analyzed

Figure S3 Multiple alignment of read pairs retrieved from gorilla "Kolo" short read archives

```
                               10        20        30        40        50        60        70        80
                       ....|....|....|....|....|....|....|....|....|....|....|....|....|....|....|....|
SVA_D & gorilla flank  CAGCTCATTGAGAACGGGCCATGATGACAATGGCGGTTTTGTGGAATAGAAAGGGGGGAAAGGTGGGGAAAAGATTGAGA
Kolo_read              -----------------------------------.................A...........................
SRR747998.115816966.2  -----------------------------------.................A...........................
SRR747998.18501377.1   -----------------------------------.................A...........................
SRR747996.91193397.1   -----------------------------------.................A...........................
SRR747995.95303290.2   -----------------------------------.................A...........................

                               90       100       110       120       130       140       150       160
                       ....|....|....|....|....|....|....|....|....|....|....|....|....|....|....|....|
SVA_D & gorilla flank  AATCGGATGGTTGCCGTGTCTGTGTAGAAAGAAGTAGACATGGGAGACTTTTCATTTTGTTCTGTACTAAGAAAAATTCT
Kolo_read              ..............T........................................-------------------------
SRR747998.115816966.2  ..............T........................................-------------------------
SRR747998.18501377.1   ..............T........................................-------------------------
SRR747996.91193397.1   ..............T........................................-------------------------
SRR747995.95303290.2   ..............T........................................-------------------------

                              410       420       430       440       450       460       470       480
                       ....|....|....|....|....|....|....|....|....|....|....|....|....|....|....|....|
SVA_D & gorilla flank  GTTCACTTGTTTATCTGCTGACCTTCCCTCCACTATTGTCCTATGACCCTGCCAAATCCCCCTCTGCGAGAAACACCCAA
SRR747998.115816966.1  ---------------------------------------------------.....a.........g.............
SRR747998.18501377.2   -----------------------------------------------------..ca.........t.............
SRR747996.91193397.2   -----------------..............c.....t.............A......................C.....
SRR747995.95303290.1   --------------..tg.cc..cc...c........t....gac...c.................T.............

                              490       500       510       520       530       540       550       560
                       ....|....|....|....|....|....|....|....|....|....|....|....|....|....|....|....|
SVA_D & gorilla flank  GAATGATCAATAAAAAAAAAAAAAAAAAAACCTTTCAGCTGGGCACAGTGGCTCACACCTGTAATCCCAGCACTGCGAGA
SRR747998.115816966.1  ......AA..G..........G.GGGGTGG.AGAGA.C.ACCTGG.CCA.C.CTTAGTA..AGGCT..TCT
SRR747998.18501377.2   a.......................G...TTAA.AA.TC...A..C.TCACT..ACT.TAGAAT.GAGATATGT
SRR747996.91193397.2   ...A.......T.............GTGT.AAAAAA.------------------------------------
SRR747995.95303290.1   .........................G...TTAA.---------------------------------------
```

The reference sequence on top of the alignment is composed of the SVA_D SINE-R consensus and the 3' flanking sequence found in the gorilla genome. The extension of the read provided by Levy et al. [1] (Kolo_read) is shown in yellow; the extension of the corresponding 3' reads in grey. Lower case indicates low sequence quality.

Additional References

1. Levy O, Knisbacher BA, Levanon EY, Havlin S. Integrating networks and comparative genomics reveals retroelement proliferation dynamics in hominid genomes. Sci Adv. 2017; 3: e1701256.
