# Supplementary material for: Phylogenomic analysis reveals splicing as a mechanism of parallel evolution of non-canonical SVAs in hominine primates
Source: Mob DNA. 2018 Sep 17;9:30. doi: 10.1186/s13100-018-0135-2 (PMC6139936; doi:10.1186/s13100-018-0135-2)
Supplement: Supplementary file 5 — Multiple alignment of the consensus sequences of SVA_D subfamilies containing the hallmark A-G co-segregating substitutions at positions 228 and 242 relative to the SVA_DR consensus. (PDF 72 kb) [file 13100_2018_135_MOESM5_ESM.pdf]

Additional file 5 **Multiple alignment of the consensus sequences of SVA_D subfamilies containing the hallmark A-G co-segregating substitutions at positions 228 and 242 relative to the SVA_DR consensus**

```
SVA_DR       CAGCTCATTGAGAACGGGCCATGATGACAATGGCGGTTTTGTGGAATAGAAAGGGGGGAAAGGTGGGGAAAAGATTGAGAAATCGGATGGTTGCCGTGTCTGTGTAGAAAGAAGTAGACA
hs_SVA_D6b   ................................................................................................................G.......
hs_SVA_D6c   ..........................................A.....................................................................G.......
pt_SVA_D5a   ................................................................................................................G.......
pt_SVA_D5b   ........................A.......................................................................................G.......
pt_SVA_D5c   ........................A.....................................................................T.................G.......
gg_SVA_D5    ........................A...............................A....................G..................................G.......
gg_SVA_D5a   .............G..........A...............................A.......................................................G.......
gg_SVA_D5b   ........................A.........A.....................A....................G..................................G.......
gg_SVA_D4    ................................................................................................................G.......
gg_SVA_D4a   ....................................................................................A...........................G.......
gg_SVA_D4b   ................................................................................................................G.......
gg_SVA_D4c   ...............................................G................................................................G.......
gg_SVA_D4d   .............G...............................G.G...G...................G........GG..............C........G..GG..G.......
gg_SVA_D4e   ...............................................G................................................................G.......
gg_SVA_D4f   ..................T............................G................................................................G.......
gg_SVA_D4g   ..................T............................G................................................................G.......
gg_SVA_D4h   ..................T............................G...................................T.....................G......G.......

SVA_DR       TGGGAGACTTTTCATTTTGTTCTGTACTAAGAAAAA-TTCTTCTGCCTTGGGATCCTGTTGATCTGTGACCTTACCCCCAACCCTGTGCTCTCTGAAACATGTGCTGTGTCCACTCAGGG
hs_SVA_D6b   ....................................-.......................................................................A...........
hs_SVA_D6c   ....................................-...........................G...........................................A...........
pt_SVA_D5a   ....................................-.......................................................................A...........
pt_SVA_D5b   ....................................-.......................................................................A...........
pt_SVA_D5c   ....................................-.......................................................................A...........
gg_SVA_D5    ..........--........................-.......................................................................A...........
gg_SVA_D5a   ..........--........................-.......................................................................A...........
gg_SVA_D5b   ..........--........................-.......................................................................A...........
gg_SVA_D4    ....................................-.......................................................................A...........
gg_SVA_D4a   ....................................-.......................................................................A...........
gg_SVA_D4b   ....................................A..................................C....................................A...........
gg_SVA_D4c   ....................................A..................................C....................................A...........
gg_SVA_D4d   .............-------................A..................................C....................................A...........
gg_SVA_D4e   ....................................A..................................C....................................A...........
gg_SVA_D4f   ....................................A..................................C....................................A...........
gg_SVA_D4g   ....................................A..................................C....................................A...........
gg_SVA_D4h   ....................................A..................................C....................................A...........
```

```
SVA_DR      TTAAATGGATTAAGGGCGGTGCAAGATGTGCTTTGTTAAACAGATGCTTGAAGGCAGCATGCTCGTTAAGAGTCATCACCACTCCCTAATCTCAAGTACCCAGGGACACAAACACTGCGG
hs_SVA_D6b  ..G.............................................................C.......................................................
hs_SVA_D6c  ..G.............................................................C.......................................................
pt_SVA_D5a  ..G.............................................................C.......................................................
pt_SVA_D5b  ..G..........................................................T..........................................................
pt_SVA_D5c  ..G..........................................................T.......................T..................................
gg_SVA_D5   ..G....................................C................................................................................
gg_SVA_D5a  ..G....................................C......T.........................................................................
gg_SVA_D5b  ..G..C.................................C.........A......................................................................
gg_SVA_D4   ..G.....................................................................................................................
gg_SVA_D4a  ..G.....................................................................................................................
gg_SVA_D4b  ..G.....................................................................C...............................................
gg_SVA_D4c  ..G.....................................................................C...............................................
gg_SVA_D4d  ..G.....................................................................................................................
gg_SVA_D4e  ..G.....................................................................C...............................................
gg_SVA_D4f  ..G.....................................................................C...............................................
gg_SVA_D4g  ..G.....................................................................C...............................................
gg_SVA_D4h  ..G.....................................................................C...............................................

SVA_DR      AAGGCCGCAGGGTCCTCTGCCTAGGAAAACCAGAGACCTTTGTTCACTTGTTTATCTGCTGACCTTCCCTCCACTATTGTCCTATGACCCTGCCAAATCCCCCTCTGCGAGAAACACCCA
hs_SVA_D6b  ...................................................................................G....................................
hs_SVA_D6c  ...................................................................................G....................................
pt_SVA_D5a  ...................................................................................G....................................
pt_SVA_D5b  ....................................A..............................................G....................................
pt_SVA_D5c  ....................................A..............................................G....................................
gg_SVA_D5   .........A..........................A..............................................G........................A...........
gg_SVA_D5a  .........A..........................A..............................................G........................A...........
gg_SVA_D5b  .........A..........................A..............................................G........................A.........T.
gg_SVA_D4   ....................................A..............................................G....................................
gg_SVA_D4a  ....................................A......................................G.......G............G.......................
gg_SVA_D4b  ....................................A..............................................G.A..................................
gg_SVA_D4c  ....................................A..............................................G.A..................................
gg_SVA_D4d  ........................T...........A..............................................G.A....................C.............
gg_SVA_D4e  ....................................A.............C................................G.A..................................
gg_SVA_D4f  ....................................A.............C................................G.A.....A............................
gg_SVA_D4g  .........................G..........A.............C................................G.A.....A............................
gg_SVA_D4h  ....................................A.............C........C.......................G.A..................................

SVA_DR      AGAATGAT
hs_SVA_D6b  ........
hs_SVA_D6c  ........
pt_SVA_D5a  ........
pt_SVA_D5b  ........
pt_SVA_D5c  ........
gg_SVA_D5   ........
gg_SVA_D5a  ........
gg_SVA_D5b  ........
gg_SVA_D4   ........
gg_SVA_D4a  ........
gg_SVA_D4b  ........
gg_SVA_D4c  ........
gg_SVA_D4d  ........
gg_SVA_D4e  ........
gg_SVA_D4f  ........
gg_SVA_D4g  ........
gg_SVA_D4h  ........
```
